# Mesoscopic analysis of drag reduction performance of bionic furrow opener based on the discrete element method

**DOI:** 10.1371/journal.pone.0293750

**Published:** 2023-11-03

**Authors:** Guomin Liu, Jiuyuan Yao, Zhen Chen, Xuekai Han, Meng Zou

**Affiliations:** 1 College of Civil Engineering, Jilin Jianzhu University, Changchun, China; 2 Key Laboratory of Bionic Engineering, Jilin University, Changchun, China; Newcastle University, UNITED KINGDOM

## Abstract

In order to study the dynamic interface mechanical behavior between soil and agricultural machinery and reveal the causes of tillage resistance, three kinds of bionic furrow opener were designed according to the characteristics of earthworm head surface curve, using the discrete element method to simulate and analyze the process of the furrow openers. The results showed that the order of ditching resistance from large to small is traditional opener, bionic corrugated opener, bionic ridgeline opener, bionic composite opener. With the same ditching speed, the drag reduction effect of the three bionic openers increases with the increase of the ditching depth. During the process of increasing the depth from 30 mm to 60 mm and 90 mm, the ditching resistance of the traditional opener increased from 11.56 N to 28.32 N and 48.61 N as well as the maximum drag reduction ratio increased from 5.58% to 7.20% and 8.93% for the bionic composite opener. With the same ditching depth, the bionic composite opener reached the highest drag reduction rate of all bionic openers when the speed is 100 mm/s, the value is 9.08%. The width of the ditch of the three bionic openers is smaller than that of the traditional opener. Bionic corrugated opener can improve the ditch height and reduce the ditch width,the corrugated structure creates a gap between the surface of the core and the particles, reducing the number of contact and contact area of the particles. The number of contact particles of the three bionic openers is smaller than that of the traditional opener. The bionic composite opener has the smallest force field and the soil disturbance caused by the core share surface is small, the soil is evenly distributed along the core surface. The discrete element simulation shows that the bionic opener can effectively reduce the ditching resistance and improve the quality of ditching, which provides a theoretical basis for subsequent research and optimization.

## Introduction

During the interaction between soil and machinery, the phenomenon of soil adhesion to the surface of soil contact components is common. Soil adhesion not only affects the working performance and quality of machinery, but also affects the normal operation [1, 2]. Among the soil machinery, the furrow opener, as an indispensable soil contact component in agricultural production, its working performance has a significant impact on agricultural production. There is inevitably soil adhesion between the soil and the furrow opener, which leads to increased resistance and energy consumption during its operation. If the resistance is too high, it may even reduce the effectiveness of trenching, leading to a decrease in crop emergence rate [3–5].

In current research, some scholars have also conducted analysis and optimization of furrow opener. Firstly, the analysis was conducted on the working conditions that affect the resistance of the furrow opener. Aiki conducted experimental analysis on different types of furrow opener,the results showed that the resistance of the furrow opener showed a significant increase with the increase of the working depth [6]. Collins analyzed the depth and speed during the working of furrow opener which showed that the influence of depth on the resistance was greater than that of speed. In addition, the results also indicate that the greater the viscosity of the soil, the greater the resistance is [7]. Allah analyzed on the furrow opener through experiments,the research showed that the speed, depth and blade insertion angle all have direct impact on the trenching resistance [8]. Sánchez-Girón compared and analyzed the effects of soil compaction and soil moisture content on the resistance. The results showed that the resistance increased with the increase of ditch depth, soil bulk density and soil moisture content. Also, the traction force of different openers had different functional relationships with soil bulk density at different depths [9]. Gebresenbet studied the effects of penetrating angle, width, depth of the furrow opener on the resistance and built a binomial model [10].

Secondly, due to the difficulty of conducting soil experiments and the variety of forms of furrow openers, some scholars have also conducted simulation studies instead of experiments. Mootaz established a finite element model of the tool through Abaqus and simulated the cutting process of the tool. The results showed that the cutting force was less affected by the cutting speed [11, 12]. Kamakara simulated the stress distribution on the tool surface and soil stress through CFD which showed that the faster the speed was, the greater the stress was [13]. Barr simulated the effects of different penetrating angle on soil disturbance of the opener and the soil using discrete element method. The simulation results were consistent with the experimental results [14]. In addition, Shmulevic, Mehari and Ying Chen did the researches on the interaction between the furrow openers and the soil using the discrete element method and the simulation and experimental results showed high accuracy [15–17]. The above research proves that the opening process of the furrow openers can be analyzed through the discrete element method and the analysis results have similarity with actual experiments.

The above research mainly focuses on the interaction between the furrow opener and the soil. Some scholars have also studied and improved the furrow opener itself. Zeng designed different types of furrow openers, which effectively reduced adhesion by changing factors such as diameter [18]. Mclees effectively improves its performance and reliability by changing traditional furrow opener materials and using ceramic materials to manufacture openers [19]. Choudhary has designed a T-shaped opener, which effectively improved the soil breaking ability but has poor soil penetration performance and high resistance.

Through the previous research, it was found that that the research on furrow openers mainly focuses on the interaction between components and soil as well as the working methods. The improvement and research on the furrow opener itself have only focused on the size improvement of traditional components, with less research on new forms of furrow openers. The existing furrow opener mainly have the disadvantages of high resistance and adhesion. This research starts from the structure of the furrow opener and combines bionic theory to design a bionic furrow opener with resistance reduction and debonding characteristics.

## Materials and methods

### Analysis of bionic prototype

From the analysis above, it can be seen that the problem faced by the furrow opener is its high adhesion during operation, which leads to excessive resistance. However, the improvement of traditional furrow openers is relatively small, so it is necessary to analyze and design furrow openers through other methods. Animals live in soil have evolved various special functions to reduce adhesion in the soil environment under the influence of natural selection. They are able to freely move in sticky and moist soil without adhering to the soil. This function has important reference value for the optimization design of the furrow openers.

For the animals, earthworms have excellent desorption and resistance reduction properties, their desorption ability mainly depends on their surface non smooth structure. Applying this non smooth structure to the design of the surface of the furrow opener can effectively reduce its surface adhesion, thereby reducing resistance [20–22].

**Fig 1:**
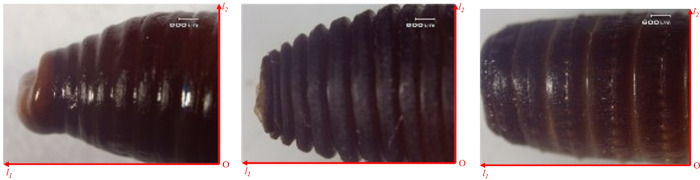
First-order macro corrugated non-smooth structure (20x magnification). (a) Contraction state. (b) Motionlessness state. (c) Stretched state.

Here, the non-smooth structure of earthworms was analyzed. Studies have shown that the corrugated body surface of earthworm has the characteristics of reducing viscosity and drag. The effect of viscosity and drag reduction of different states of the body from large to the small is: contraction state, motionlessness state, stretched state. The effect of viscosity and drag reduction of the head is more obvious than that of the body. Therefore, the head contraction state of earthworm has the best viscosity and drag reduction effect. The corrugated surface structure of earthworm is shown in Fig 1.

The coordinate points of the earthworm head soil-entering curve were obtained by the drawing software, the coordinate curves were fitted by the MATLAB software. First, the soil-entering part of the front part of the contraction curve of the earthworm head was fitted. The curve equation is as follows:

$$l_{1}=0.00001825l_{2}{}^{3}+0.003418l_{2}{}^{2}+0.2277l_{2}$$ (1)

Where *l*_*1*_ and *l*_*2*_ represent the length. Then the corrugated structure of the middle and rear part of the contraction curve of the earthworm head was fitted. The curve equation is as follows:

$$l_{1}=sin(0.2856l_{2})$$ (2)

Where *l*_*1*_ and *l*_*2*_ represent the length.

The regression coefficients *R*^2^ of the two equations after fitting are both greater than 0.99, with a higher degree of fitting, which makes the application of the bionic curve more reliable.

### Design of bionic opener

#### Traditional opener

**Fig 2:**
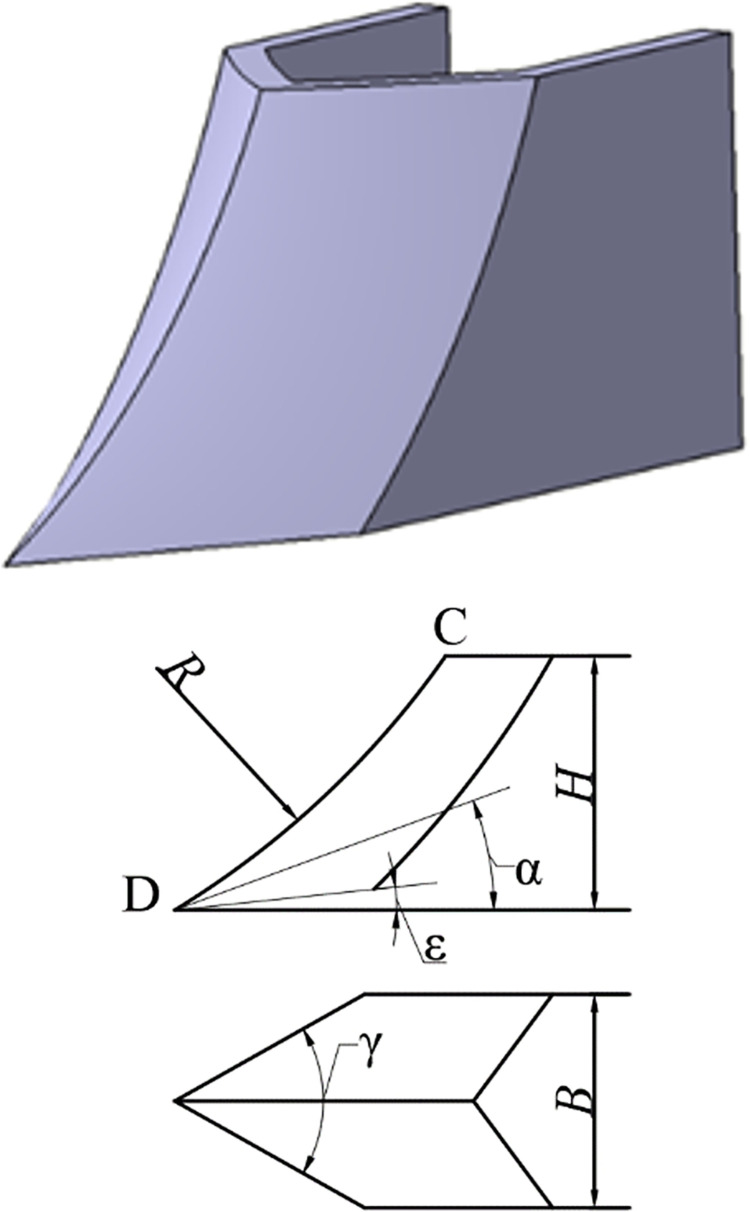
Main parameters of core structure. (a) Furrow opener. (b) The main parameters.

The paper mainly studies the contact behavior between the opener core surface and the soil interface. In order to ensure the feasibility of comparing different openers, the main structural parameters are the same, only the surface of the opener is optimized. The basic parameters of all openers are unified, the soil-entering clearance angle ε = 0°, the height H = 110 mm, the width B = 100 mm, the oblique cutting angle γ = 60°. The soil-entering angle of the traditional opener is α = 20°, the ridgeline radius R = 250 mm. The main parameters of the traditional opener and its core structure are shown in Fig 2.

#### Bionic opener

According to the structural shape of the corrugated body surface of earthworm, combined with the actual action process between the opener and the soil, the corrugated surface structure of earthworm is applied to the core surface of the opener to design bionic opener.

**Fig 3:**
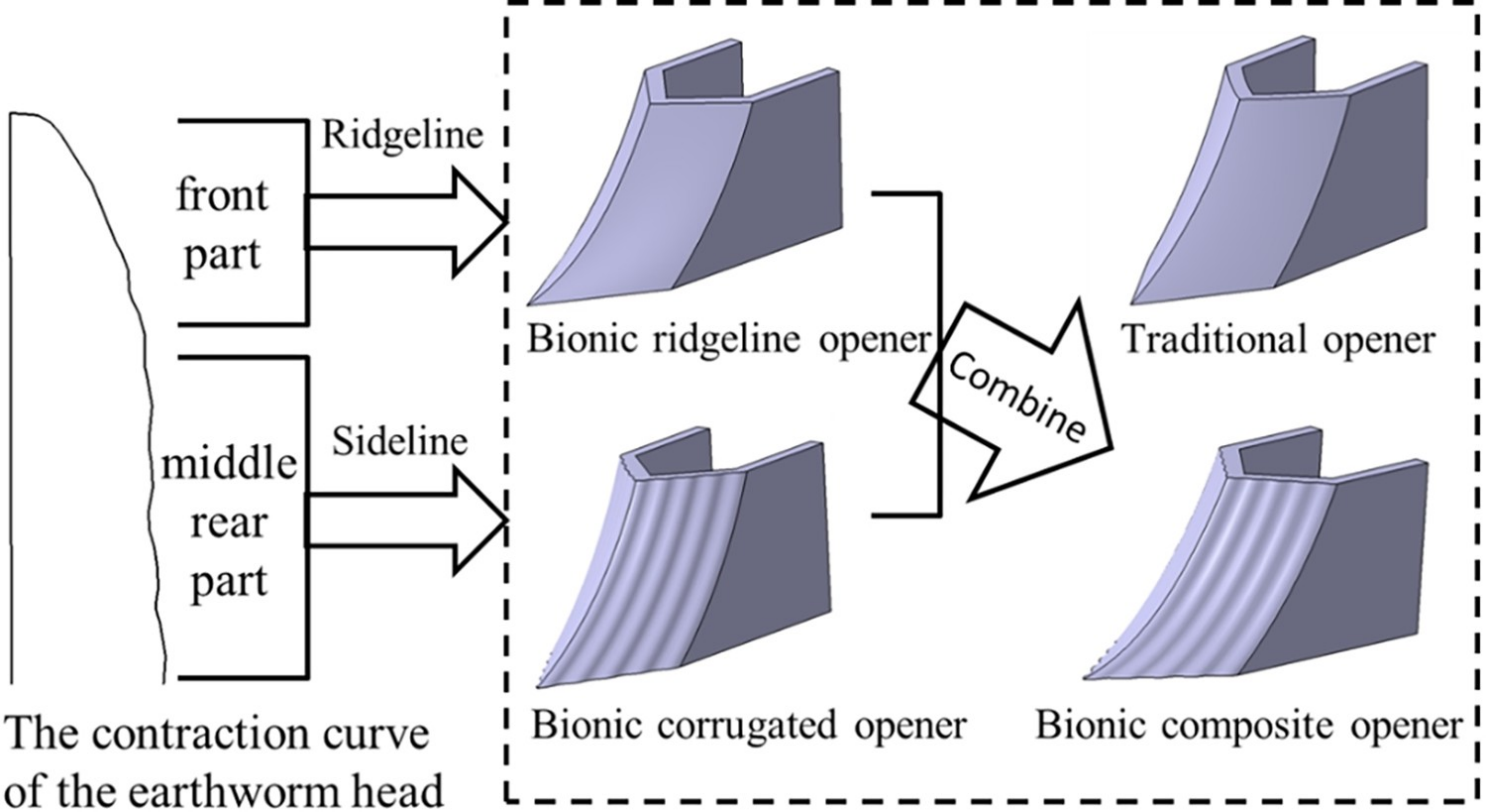
Bionic opener design diagram.

The head contraction state of earthworm has the most obvious viscosity and drag reduction effect. According to the size of the opener, the front part of the curve of earthworm head was extracted and applied to the core ridgeline to design the bionic ridgeline opener. The middle and rear part of the curve of earthworm head was extracted and applied to the core sideline to design the bionic corrugated opener. As shown in Fig 3.

**Fig 4:**
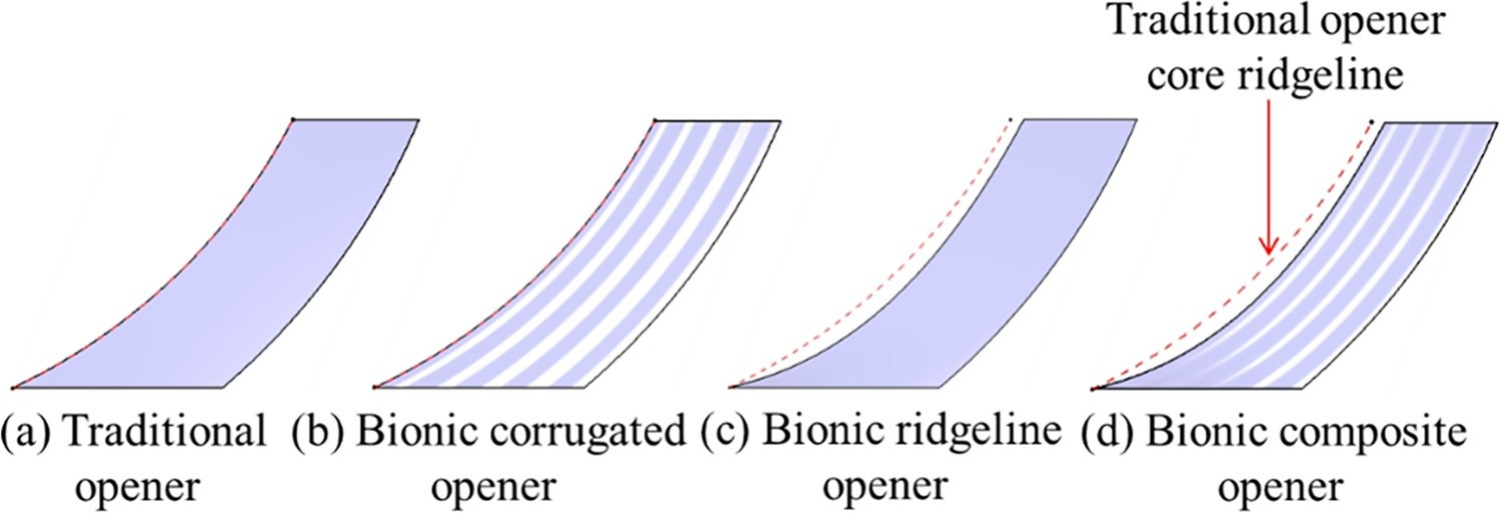
Comparison of core surface of four different openers.

The soil-entering angle of the bionic ridgeline opener is 15°, the curvature of the core ridgeline increases gradually from bottom to top. The core sideline of the bionic corrugated opener exhibits a corrugated shape extending upward along the surface of the core. Combining the two characteristics of bionic ridgeline and bionic corrugation to design the bionic composite opener, as shown in Fig 3. In order to facilitate the intuitive comparison, the comparison of the core surface of the four different openers is given, as shown in Fig 4.

### Discrete element simulation model

In order to analyze the working resistance and ditching effect of the opener and the law of soil movement, EDEM software was used to simulate the four kinds of opener. The simulation results of traditional opener, bionic ridgeline opener, bionic corrugated opener and bionic composite opener were compared and analyzed under different ditching depth and speed conditions. Macroscopic analysis the drag reduction effect and ditching effect of different openers; Microcosmic analysis the number of contact particles in contact with the core surface of the opener, the motion track, the response law of the contact force field.

#### Simulated particle parameters

**Fig 5:**
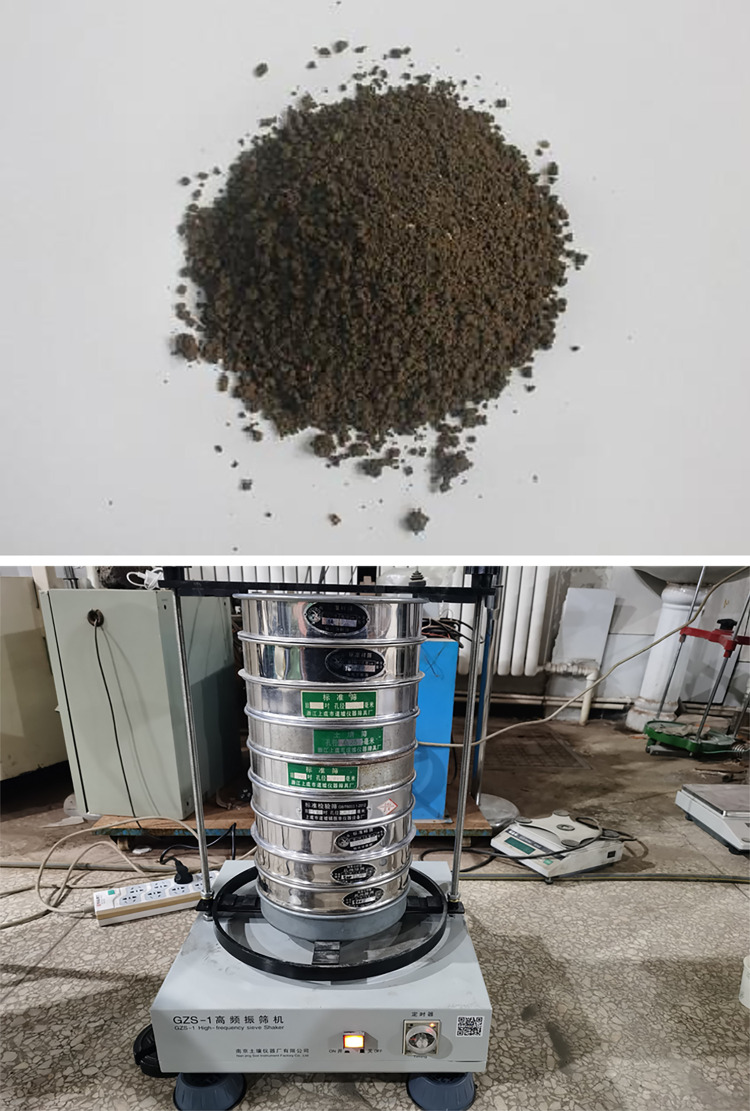
Soil testing. (a) Testing soil. Testing equipment.

To ensure consistency between the simulation and actual situation, the soil particle parameters are calibrated here, the soil is selected as the planting soil as shown in Fig 5(A). Firstly, calibrate the particle size distribution and use a high-frequency vibrating screen as shown in Fig 5(B) to calibrate the particle size distribution.

Through experiments, the soil particle size distribution is obtained as shown in Table 1. According to the data, soil particles are mainly composed of five ranges of particle size. There are fewer particles with a diameter of 3 mm or more, which may not be considered in the simulation process. The number of other four particle ratios are 5:3:2:2. The shape of the particles in the soil is very different and extremely complex, mainly including nuclear, columnar and blocky structures. In order to simulate the soil environment, the simulated particles are approximately composed of different types of particles, including single-sphere particles, double-sphere particles and three-sphere particles. As well, during the simulation process, the smaller the particle size is, the longer the simulation time required. Moreover, appropriately enlarging the particle size has little impact on simulation experiments. Therefore, in the process of generating particles, appropriately enlarging the particle size can effectively improve simulation efficiency.

**Table 1:** Particle size distribution.

Particle diameter /mm	Above 3	2–3	1–2	1–0.5	Below 0.5
Particle proportion /%	2.49	40.46	24.39	16.30	16.36

As the complex shape of soil particles, four different types of particles are set based on their size distribution and shape, namely the four particles of 1.5 mm single-sphere, 1.5 mm double-sphere, 1.5 mm three-sphere, 1mm single-sphere were generated synchronously according to the ratio of 5:3:2:2. The size of the particles are all radius. In addition, particles with a radius of 3 mm are set at the bottom layer for the bottom soil. These particles are only used for the construction of the soil tank and do no participate in the function of the furrow opener. Therefore, larger particles are selected to improve simulation efficiency.

**Fig 6:**
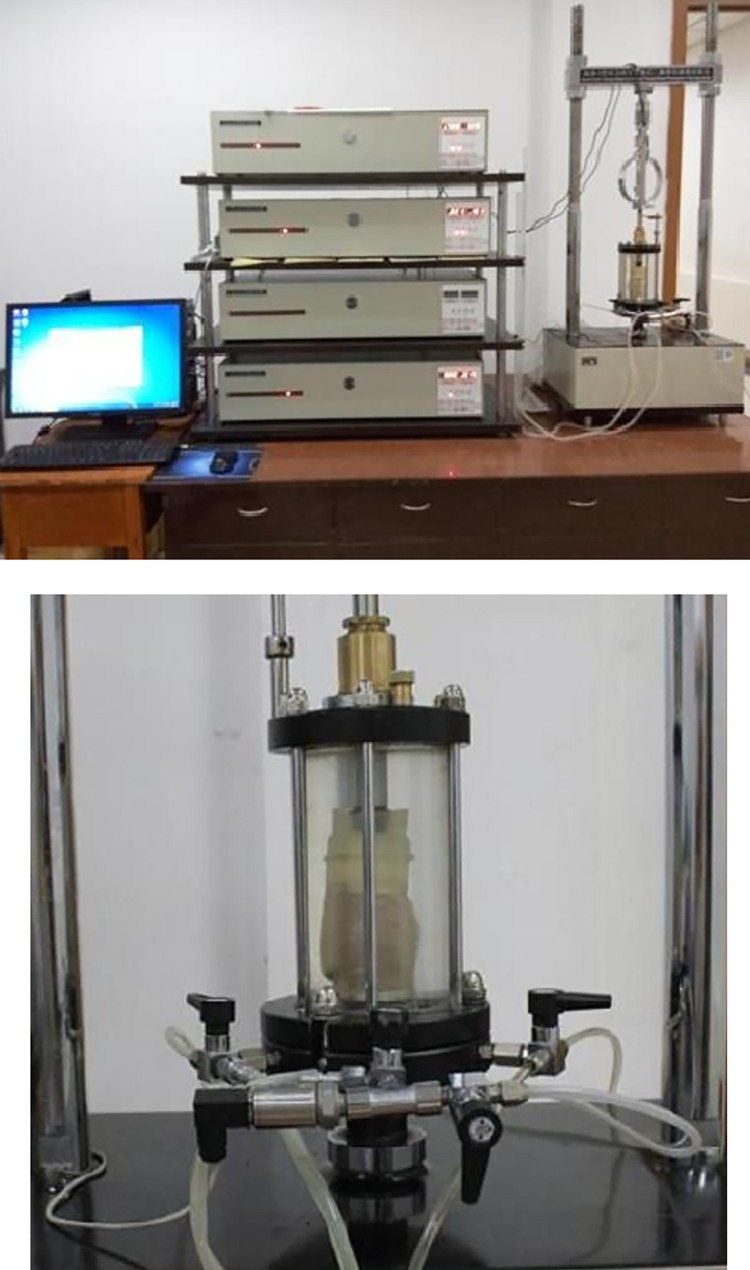
Parameter testing. (a) Triaxial test bench. (b) Testing process.

In addition to particle size and distribution, the required soil particle elastic modulus, Poisson’s ratio and shear modulus for simulation should be tested, as shown in Fig 6.

The soil parameters obtained from the experiment are summarized as shown in Table 2.

**Table 2:** Experiment results.

	Elastic modulus(MPa)	Poisson’s ratio	Shear modulus(MPa)
Experiment result	2.92	0.38	1.05

Other parameters such as the density of soil tank material and contact parameters are obtained through consultation [23–25]. The particle parameters of the established soil particles are shown in Table 3.

**Table 3:** Parameters value.

Parameters	Parameter value
Parameters: soil bin dimensions (length×width×height) / (mm)	1400×400×400
Ditching speed *v* / (mm∙ s^-1^)	50–900
Ditching depth *h* /mm	30–90
Density of soil particles ρ_1_/ (kg∙m^-3^)	1300
Density of Steel ρ_1_/ (kg∙m^-3^)	7800
Poisson’s ratio of Steel *v*_1_	0.3
Shear modulus of Steel G_1_ /Pa	7.27×10^10^
Coefficient of restitution between the soil and soil *e*_*1*_	0.13
Coefficient of rolling friction between the soil and soil *e*_*2*_	0.22
Coefficient of static friction between the soil and soil *e*_*3*_	0.4
Coefficient of restitution between the soil and Steel *f*_*1*_	0.15
Coefficient of rolling friction between the soil and Steel *f*_*2*_	0.32
Coefficient of static friction between the soil and Steel *f*_*3*_	0.5
Acceleration of gravity g / (m∙s^-2^)	9.81
Normal stiffness per unit area (N∙m^-3^)	19000
Shear stiffness per unit area (N∙m^-3^)	14000
Critical normal stress (Pa)	55000
Critical shear stress (Pa)	29000
Bonded disk radius/(mm)	1.5–3

**Fig 7:**
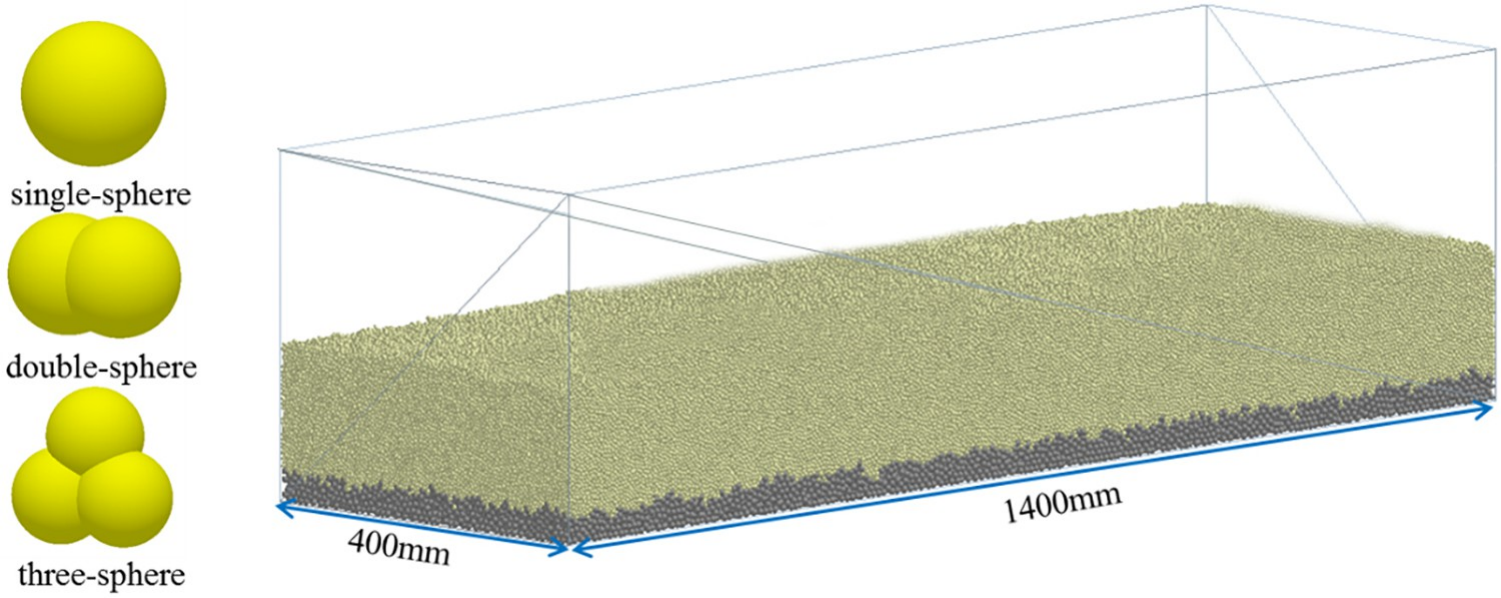
EDEM simulation model.

Input the above particle parameters into the discrete element software, the generated particles and soil slots are shown in Fig 7.

### Verification of soil particle accuracy

In order to ensure consistency between the simulation and the real environment, experiments are conducted on the particles obtained during the simulation process. Comparing it with actual soil parameters to ensure the accuracy of the simulation experiment.

**Fig 8:**
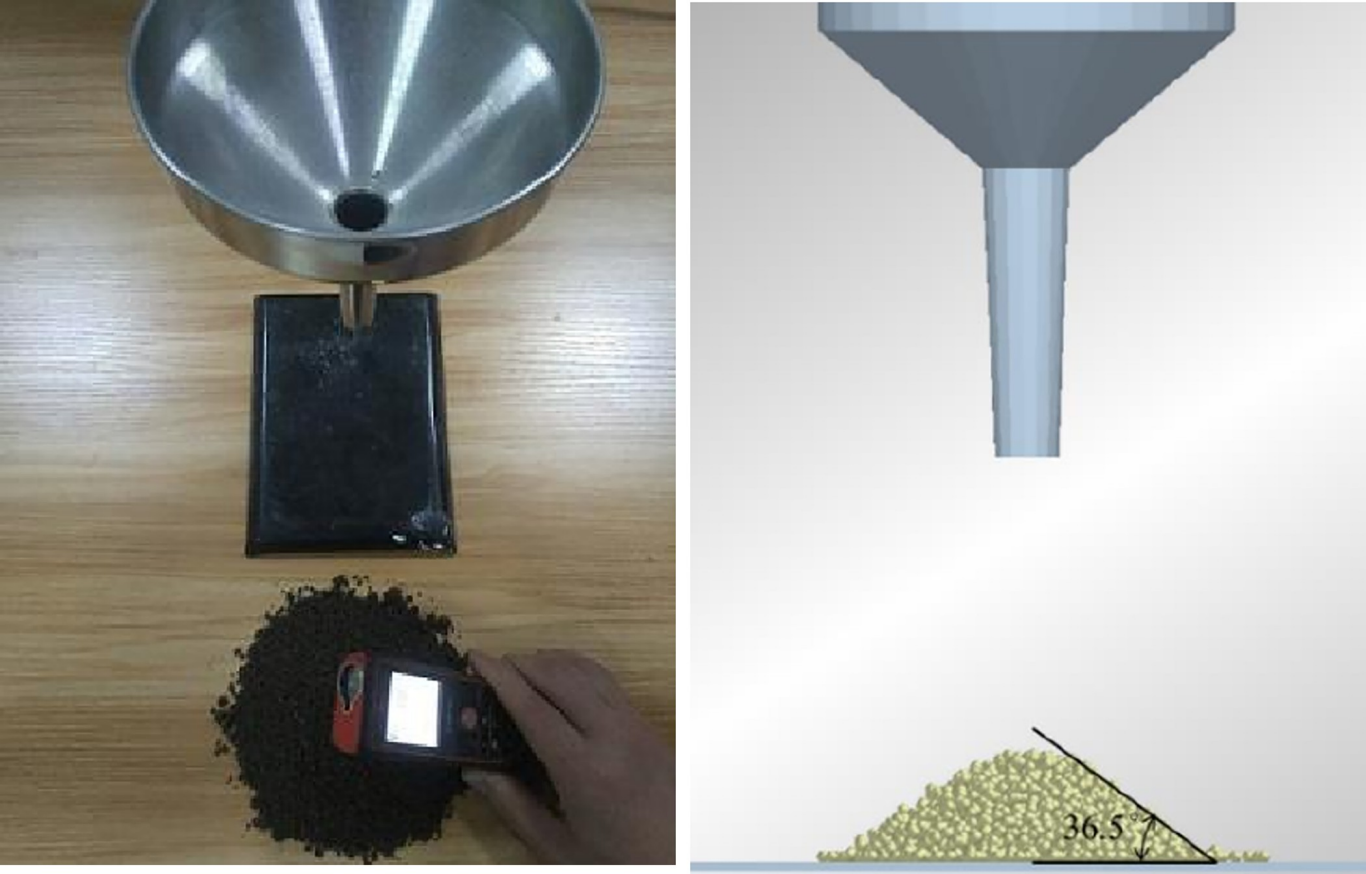
Parameter comparison experiment. (a) Real soil testing. (b) Simulated soil testing.

Here, a stacking angle test was conducted to test and compare the collision recovery coefficient, static friction coefficient and dynamic friction coefficient between soil particles. The experimental process and simulation process are shown in Fig 8.

The soil particles parameters obtained from the measurement of soil particles and simulation parameters are shown in Table 4. From the comparative parameter analysis, it can be seen that the actual soil particles sampled are relatively close to the parameters generated during the simulation process which leads that the simulated soil and actual soil has a high similarity between each other. This type of particle can be used as a substitute for physical testing to analysis.

**Table 4:** Parameters comparison.

	Experimental Value	Simulation Value	Relative Error
Stacking Angle(°)	35.9	36.5	1.6%
Collision Recovery Coefficient	0.12	0.13	7.6%
Static Friction Coefficient	0.37	0.40	7.5%
Rolling Friction Coefficient	0.24	0.22	9%

## Results and analysis

### Drag reduction analysis

**Fig 9:**
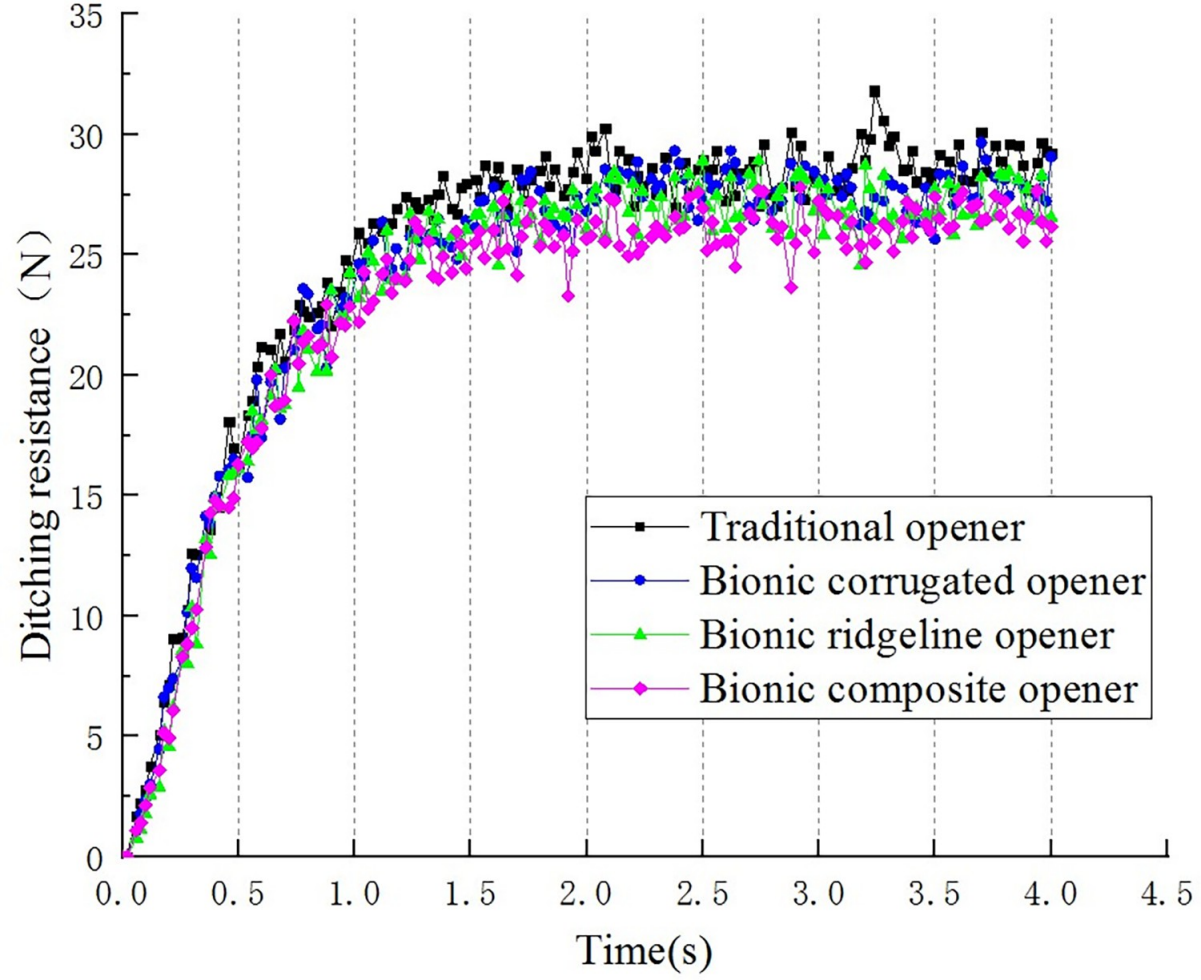
Comparison of original data of ditching resistance of four openers.

The total force of the opener in the y direction is defined as the forward resistance of the opener, the forward resistance of different openers is compared. Fig 9 is a comparison diagram of the ditching resistance curves of four openers at a speed of 300 mm/s and depth of 60 mm.

As can be seen from Fig 9, the motion of the opener is mainly divided into two stages. The first stage is the soil entry stage of the opener(0–1.5 s). The surface of the core gradually breaks into the soil, the working resistance is continuously increasing. The second stage is the ditching stabilization stage(1.5 s-4s), the working resistance remains stable. The average value of the ditching resistance in the stable stage was taken as the comparative value of the ditching resistance of different openers for analysis.

**Fig 10:**
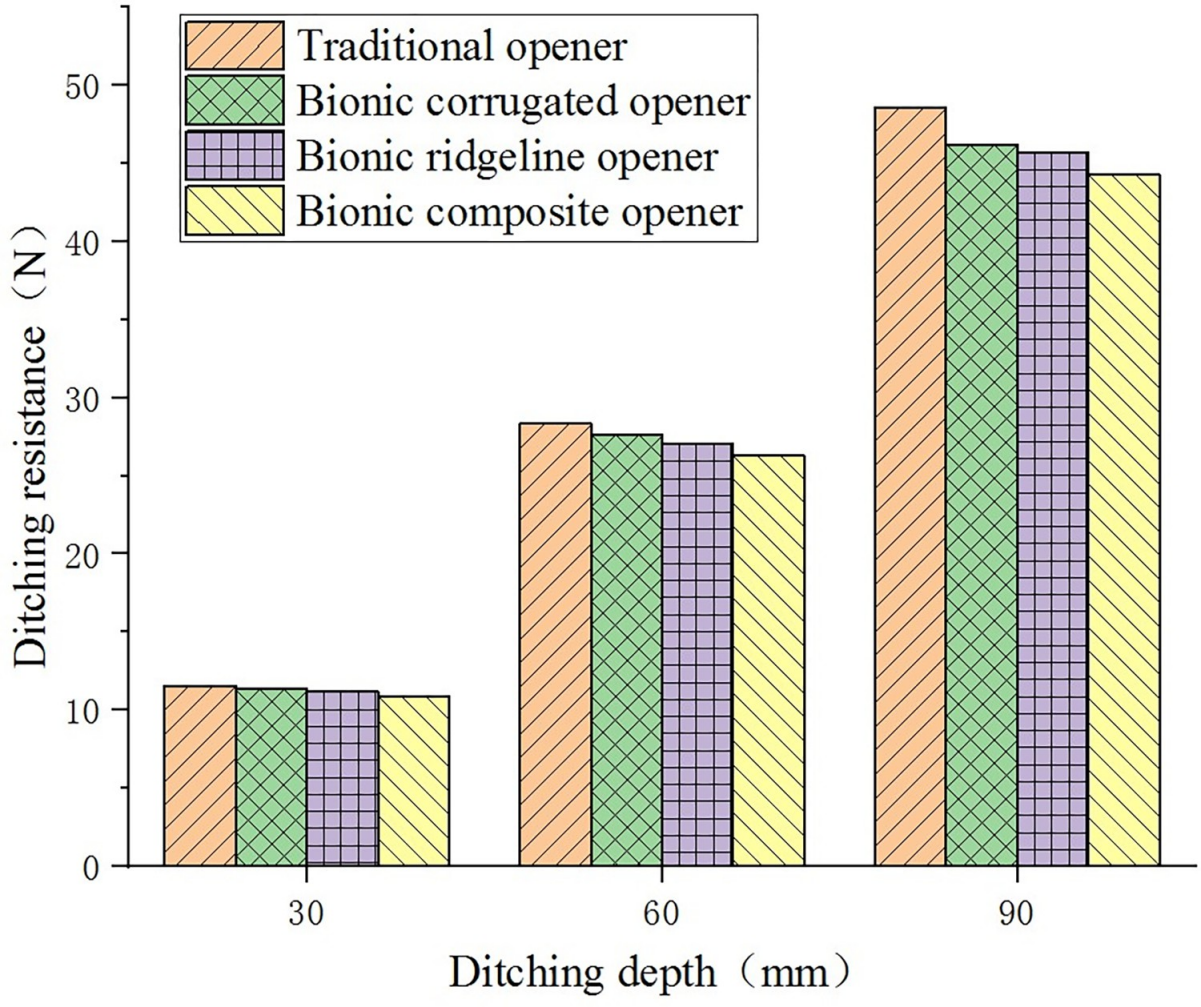
Effect of ditching depth on ditching resistance.

Based on the analysis of the drag reduction of different types of openers, the influence of the ditching speed and ditching depth of the opener on the ditching resistance should be understood. Fig 10 shows the ditching resistance values of the four openers at the ditching depth of 30 mm, 60 mm and 90 mm when the speed is 300 mm/s.

It can be seen from Fig 10 that as the ditching depth increases, the ditching resistance increases. Under the same ditching depth conditions, the ditching resistance of each opener from large to small is: traditional opener, bionic corrugated opener, bionic ridgeline opener, bionic composite opener. During the process of increasing the depth from 30 mm to 60 mm and 90 mm, the ditching resistance of the traditional opener increased from 11.56 N to 28.32 N and 48.61 N. Comparing the drag reduction ratios of the three bionic openers with respect to the traditional opener, the drag reduction ratio is shown in Table 5.

**Table 5:** Drag reduction ratio.

Ditching depth /mm	Bionic corrugated opener	Bionic ridgeline opener	Bionic composite opener
30	1.98%	3.4%	5.58%
60	2.4%	4.52%	7.2%
90	4.9%	5.92%	8.93%

It can be seen from Table 5 that the drag reduction effect of the three bionic openers increases with the increase of the ditching depth. The drag reduction effect of the three openers from small to large is: bionic corrugated openers, bionic ridgeline openers and bionic composite openers. The bionic composite opener achieves the maximum drag reduction ratio of 8.93% at the depth of 90 mm.

**Fig 11:**
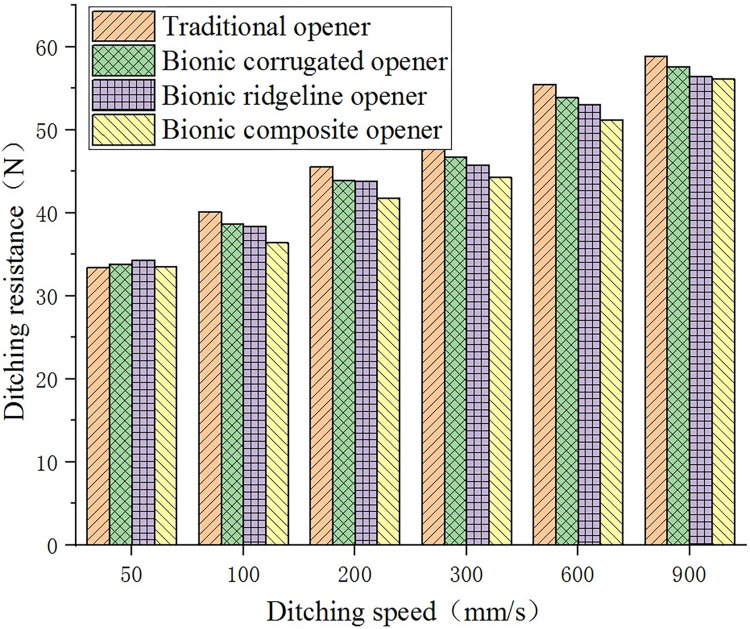
Effect of ditching speed on ditching resistance.

Fig 11 shows the ditching resistance values of the opener at the ditching speed of 50 mm/s, 100 mm/s, 200 mm/s, 300 mm/s, 600 mm/s and 900 mm/s, respectively, when the ditching depth is 90 mm. It can be seen from Fig 11 that the ditching resistance of the opener increases with the increase of the ditching speed. Except for 50 mm/s, the ditching resistance of each opener keeps the same order from large to small. The traditional opener has the largest ditching resistance, the bionic composite opener has the smallest ditching resistance. The drag reduction ratio of each bionic opener is shown in Table 6.

**Table 6:** Drag reduction ratio.

Ditching speed /mm/s	Bionic corrugated opener	Bionic ridgeline opener	Bionic composite opener
50	+	+	+
100	3.40%	4.12%	9.08%
200	3.60%	3.82%	8.37%
300	3.95%	5.92%	8.93%
600	2.86%	4.32%	7.61%
900	2.12%	4.67%	4.21%

The drag reduction ratio of the three bionic openers has different trends with the increase of the ditching speed. The drag reduction ratio of the bionic corrugated opener increases first and then decreases with the increase of the ditching speed; the maximum drag reduction ratio is 3.95% at the speed of 300 mm/s. The drag reduction ratio of the bionic ridgeline opener remains basically the same with the increase of the ditching speed. The drag reduction ratio of the bionic composite opener decreases with the increase of the ditching speed, the maximum drag reduction ratio is 9.08% at the speed of 100 mm/s.

The corrugated structure of the bionic corrugated opener reduces the contact area between the core surface and the soil without changing the curvature of the surface of the core. The bionic ridgeline opener provides a smaller soil-entering angle and improves the soil entry performance. The curvature of the ridgeline increases gradually, which is gentler than the ridgeline of the traditional opener, thus reducing the ditching resistance. The bionic composite opener has the minimum ditching resistance.

### Analysis of ditching quality

According to the performance evaluation index of the opener, the analysis of the quality of the opener mainly includes soil disturbance analysis and soil backing analysis. Due to the limitation of discrete element simulation, we do not consider the stability and passability of ditching. The aim of the research and optimization of the ditching quality is to reduce the soil disturbance and increase the soil backing rate.

#### Analysis of soil disturbance

**Fig 12:**
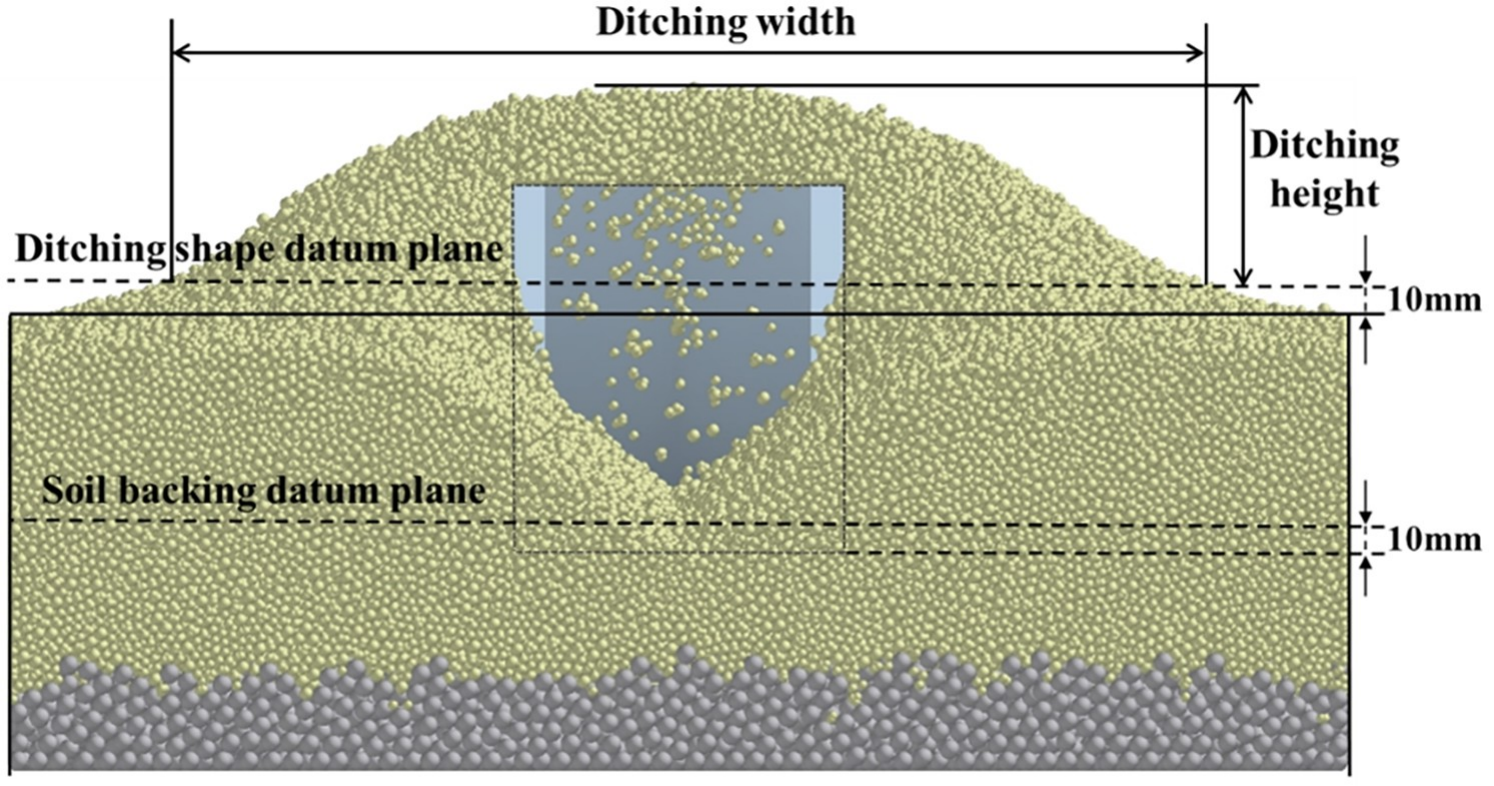
The simulated ditching shape and parameters.

Under the condition that the main structural parameters such as the height and width of the four openers are the same, the ditching situation is analyzed. Under the condition of 100 mm/s speed and 60 mm depth, the ditching width and the ditching height were measured to compare the overall disturbance state of soil caused by the four openers. The simulated ditching shape and parameters are shown in Fig 7 and Table 3. In order to reduce the error and facilitate the measurement, a datum plane of 10 mm from the surface of the soil layer was established to measure the ditching width and the ditching height above the datum plane. After the ditching width value is reduced by 200, the ditching height is drawn in the same histogram, as shown in Fig 12.

**Fig 13:**
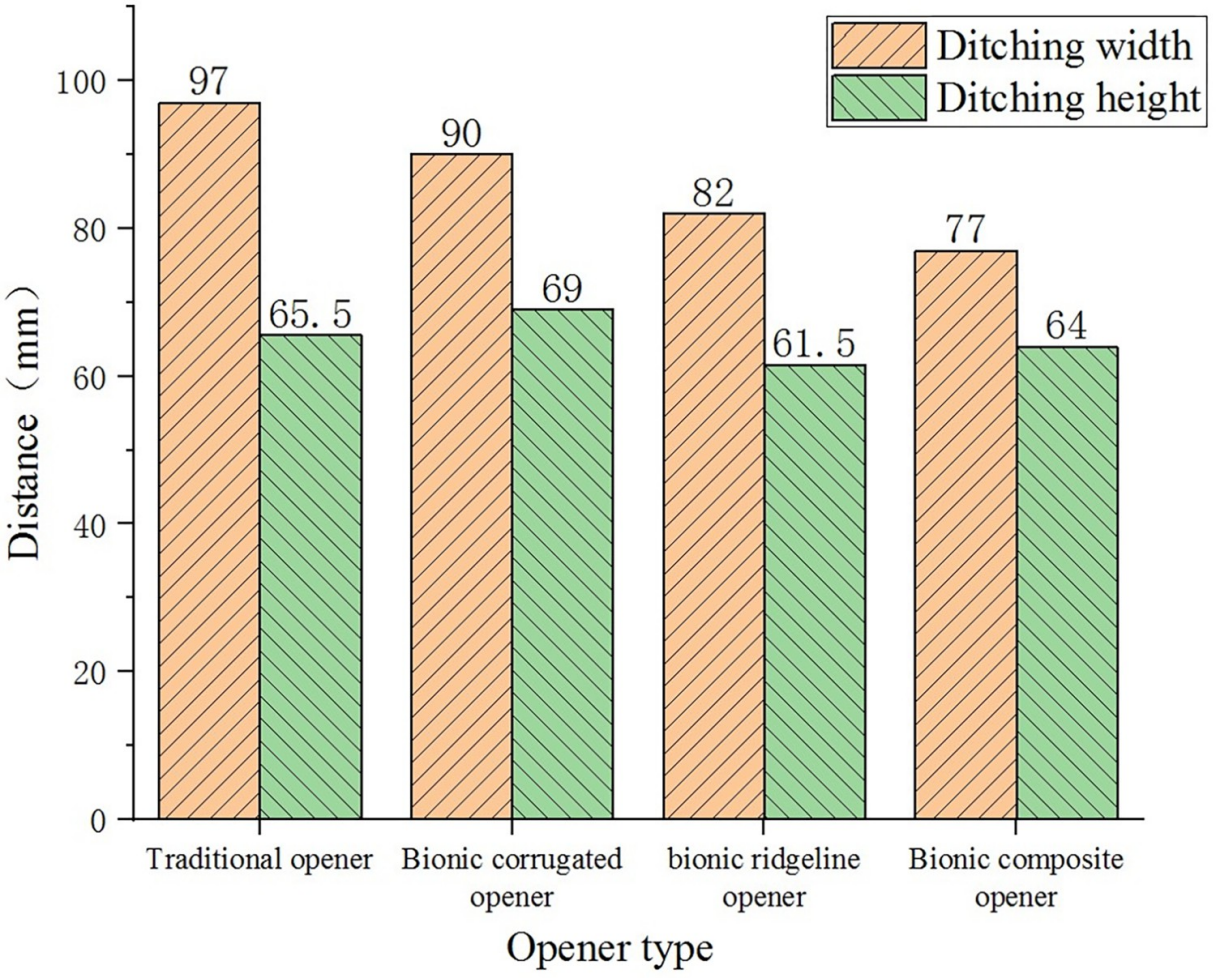
Specific values of ditching parameters.

It can be seen from the simulation results that the bionic corrugated opener has the largest ditching height and a smaller ditching width in Fig 13. The corrugated structure can make the soil move vertically upward along the corrugated surface, increasing the ditching height and reducing the ditching width. The bionic ridgeline opener can effectively reduce the ditching width and the ditching height. The bionic composite opener is the coupling of bionic ridgeline and bionic corrugation. It has the smallest ditching width and a smaller ditching height. The ditching width of the three bionic openers is smaller than that of the traditional opener. This allows the opener to complete the ditching while reducing the disturbance to the surrounding soil.

### Analysis of soil backing

**Fig 14:**
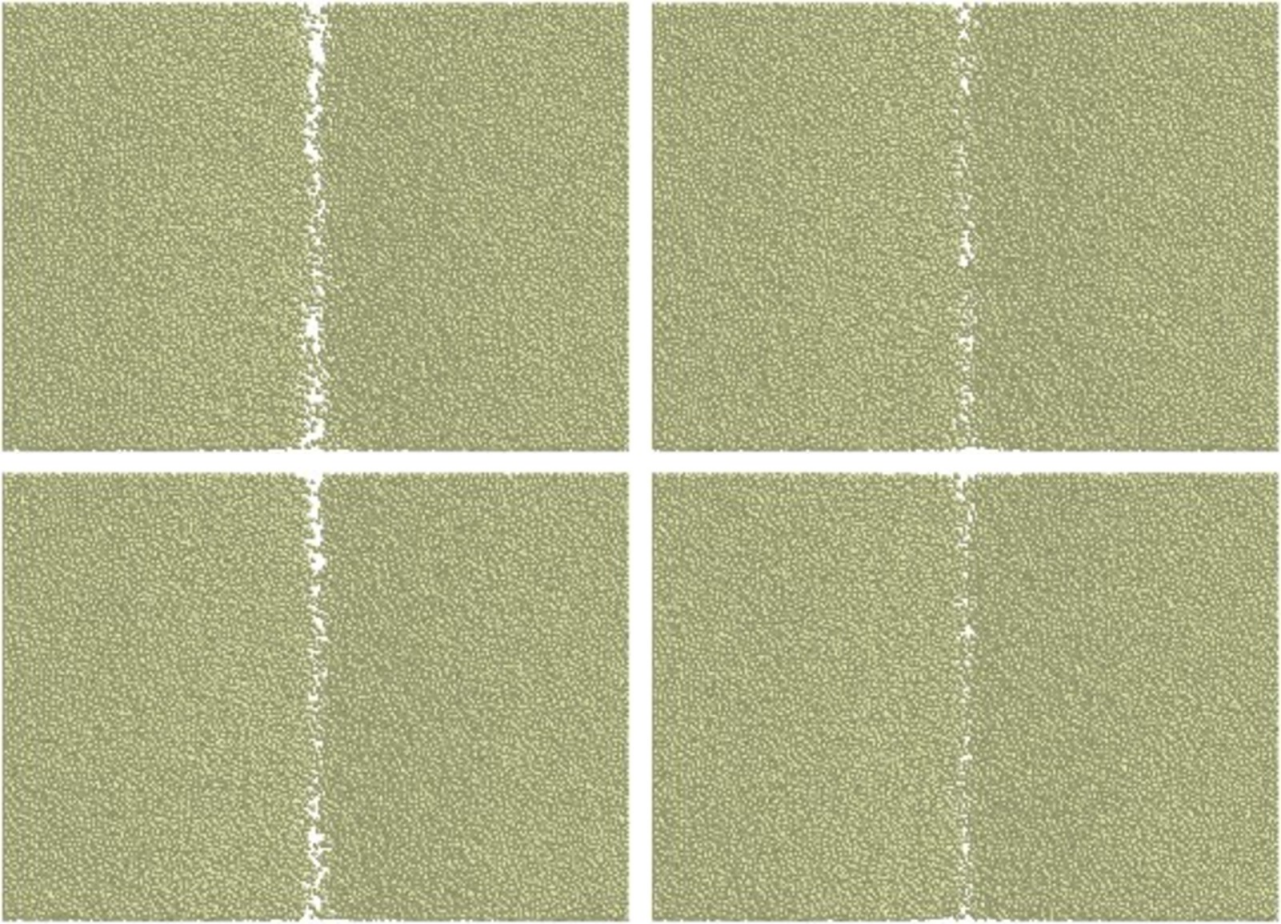
Top view of soil backing. (a) Traditional opener. (b) Bionic corrugated opener. (c) Bionic ridgeline opener. (d) Bionic composite opener.

The four openers have the same width and size, the soil cover condition is basically the same. In order to make a more intuitive comparison, lift the bottom surface of the opener up by 10 mm to establish the soil backing datum plane (Fig 12). The soil particles under the datum plane were hidden to observe the soil backing condition, as shown in Fig 14. It can be seen that the three bionic openers have better soil backing effect than the traditional openers, the bionic corrugated opener and bionic composite opener which with corrugated structure are more obvious. The corrugated structure can promote the upward movement of soil particles and increase the ditching height, thereby promoting soil back to soil.

### Analysis of contact behavior

Microscopic analysis of the interface between the opener and the soil can better understand the contact effect between the opener and the soil. EDEM software can quantitatively analyze the number of particles in contact with the soil during the working process of the opener, can show the movement track of the soil particles, the microscopic disturbance of the soil and the contact force field.

#### The number of contact particles

**Fig 15:**
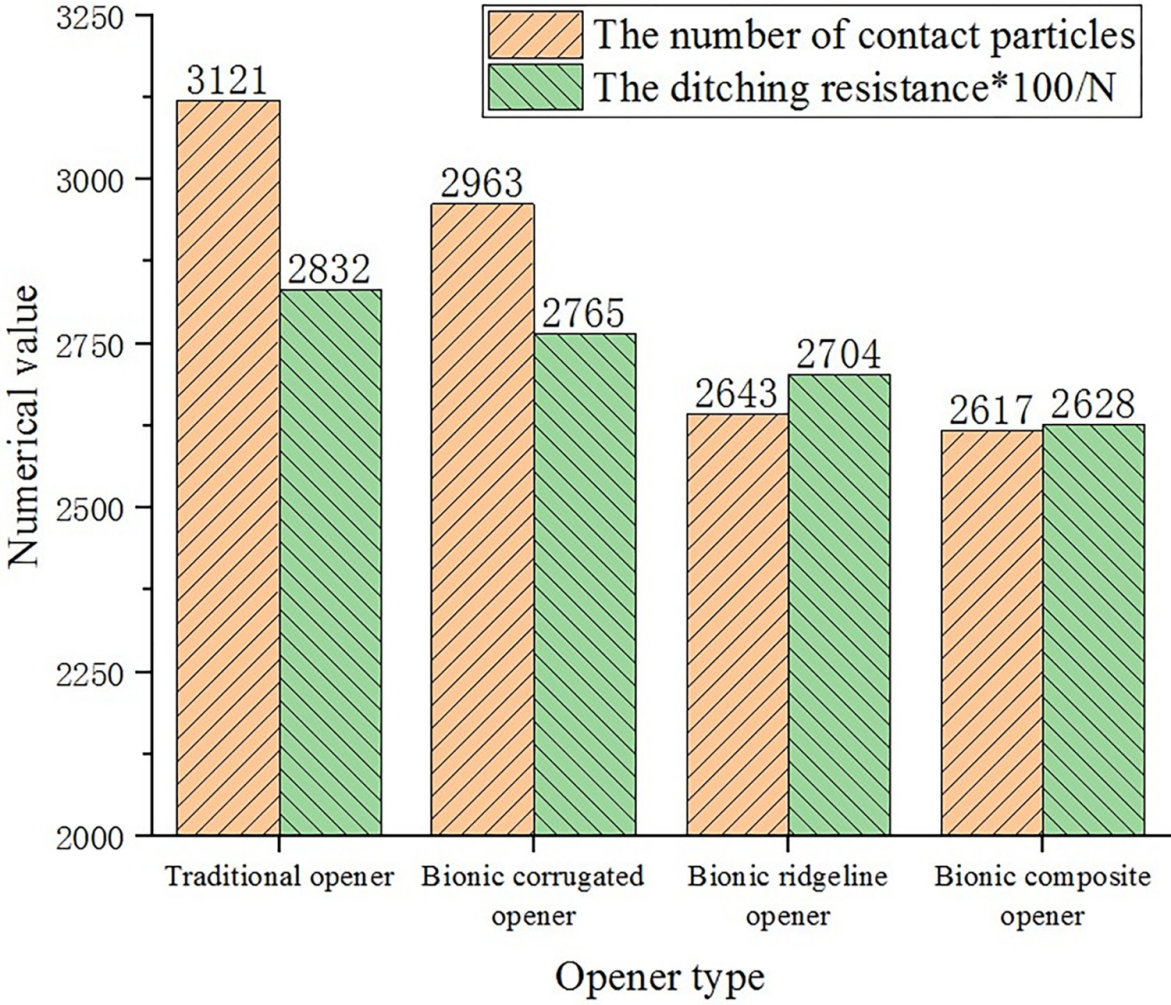
Particle contact number and ditching resistance diagram.

At a speed of 300 mm/s and a depth of 60 mm, the number of contact particles between the core surface of the opener and the soil particles during the working stable stage was analyzed. Fig 15 shows the number of contact particles and the ditching resistance of the opener recorded in the discrete element simulation.

It can be seen from Fig 15 that the number of contact particles of the traditional opener is the largest, the number of contact particles of the three bionic openers is smaller than that of the traditional opener. The ditching resistance has the same tend as the number of contact particles, both have a positive correlation.

**Fig 16:**
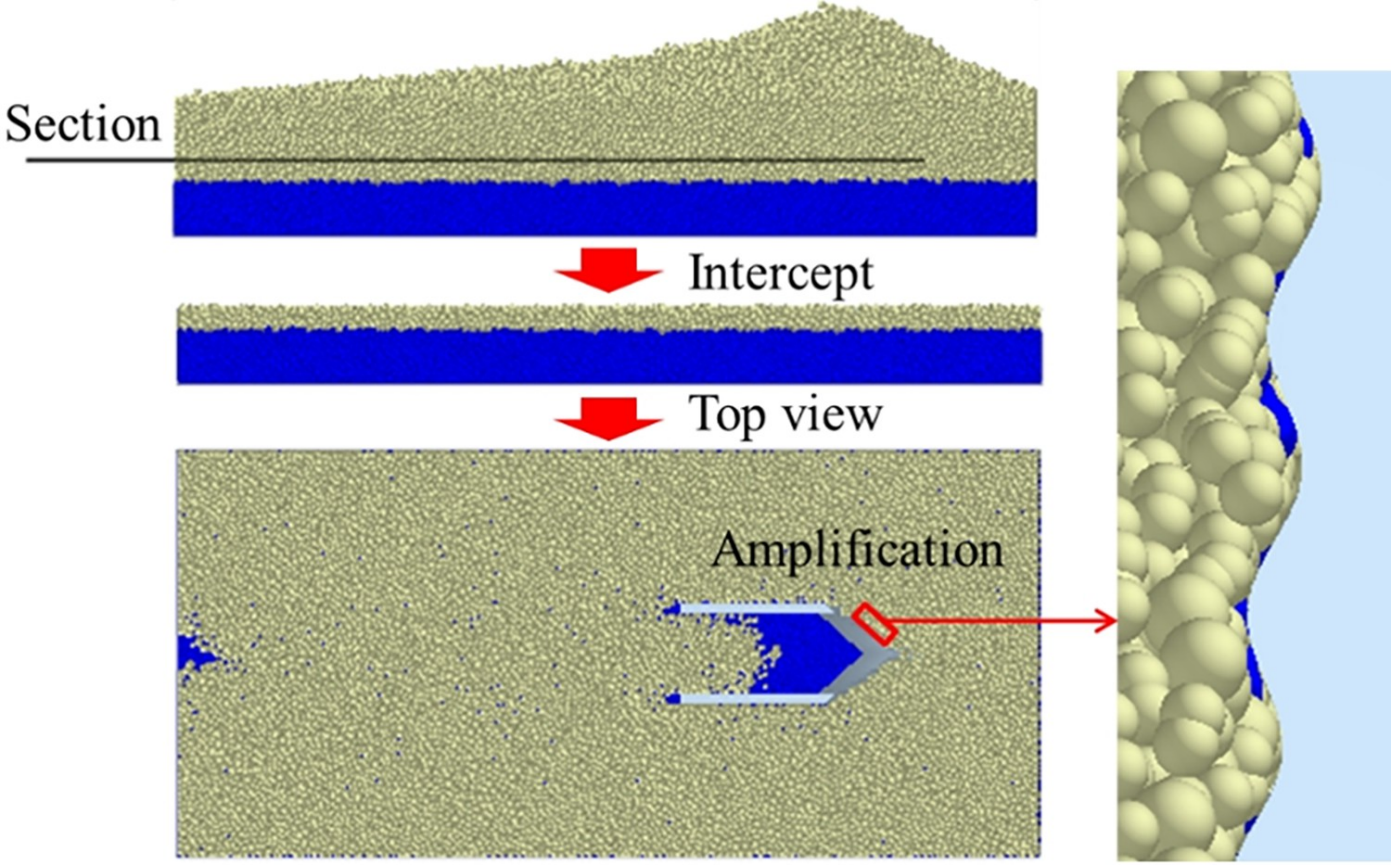
The interface between the corrugated core surface and the soil.

The bionic ridgeline opener reduces the surface area of the core surface due to the change of the curvature of the ridge line, thereby reducing the number of contact particles between the surface of the core and the soil. The concave-convex corrugated core surface of the two corrugated type openers makes the surface of the core not completely in contact with the soil particles, leaving a gap at the contact interface to produce an air film. The contact area between the surface of the core and the soil is reduced, thereby having a smaller number of contact particles. The interface between the surface of the corrugated core and the soil is shown in Fig 16.

### Movement track of soil particles

**Fig 17:**
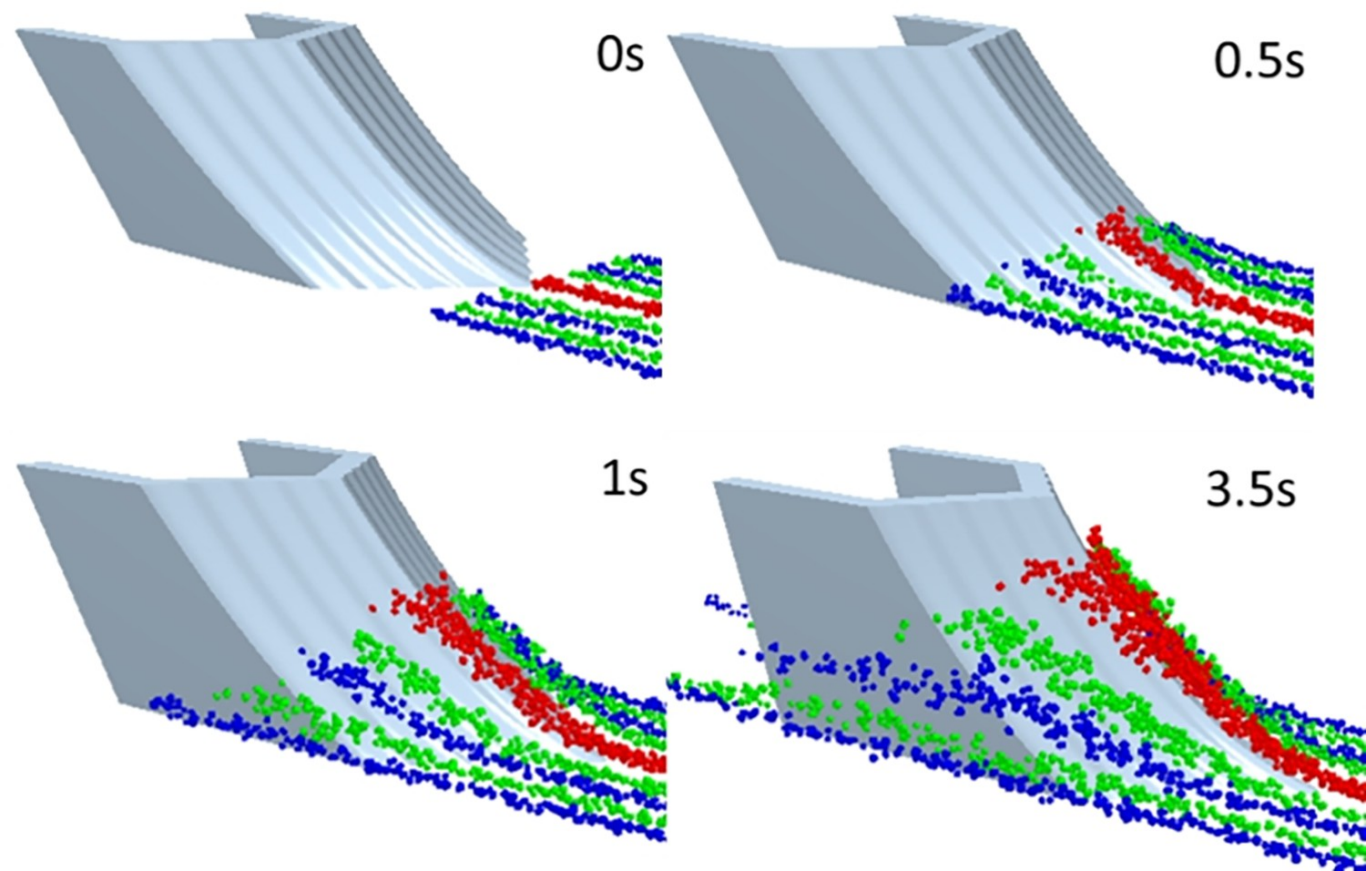
The track of particle beam.

The microscopic comparative analysis was carried out by comparing the relative motion of the opener and the soil during the ditching process. After the simulation is completed, the time is adjusted to the starting moment, the particles are selected into 9 different particle beams, the particle beam is in a straight-line state at this time. Adjust the simulation time to obtain the particle beam track at different times. The track of particle beam changes with time is shown in Fig 17.

**Fig 18:**
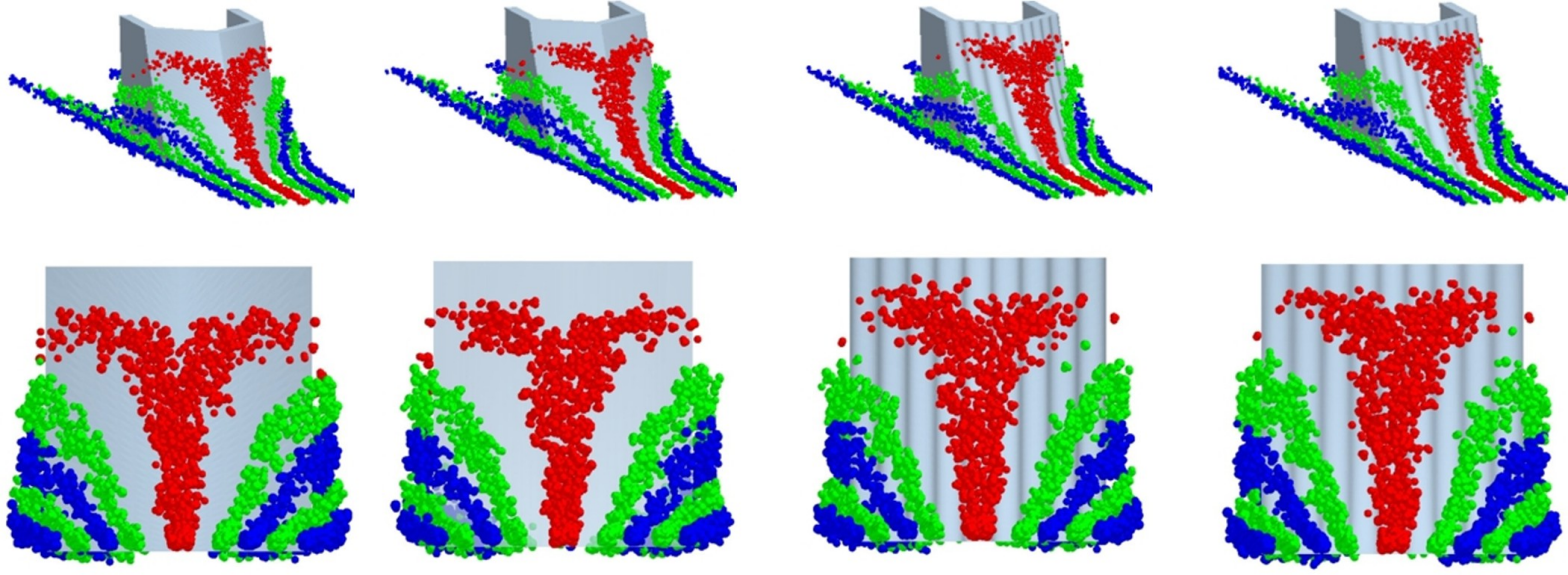
Soil particle motion track. (a) Traditional opener. (b) Bionic ridgeline opener. (c) Bionic corrugated opener. (d) Bionic composite opener.

The time was adjusted to the stable stage of ditching (3.5 s), the movement track of the soil particles was observed by the change of the particle beam, as shown in Fig 18. It can be seen from Fig 18 that the track of the soil particles changes less than that of the traditional one. The particle track of the bionic corrugated opener and bionic composite opener with corrugated structure is steeper than that of the traditional one. The corrugated structure can guide the particles to run in the vertical direction, such that the force applied by the particles to the surface of the core is relieved from the tip end of the core to the entire surface of the core, thereby increasing the travel of the particles. This is also the reason why the number of contact particles is reduced less than that of the traditional opener, also the reason why the ditching width is reduced.

### The contact force field

**Fig 19:**
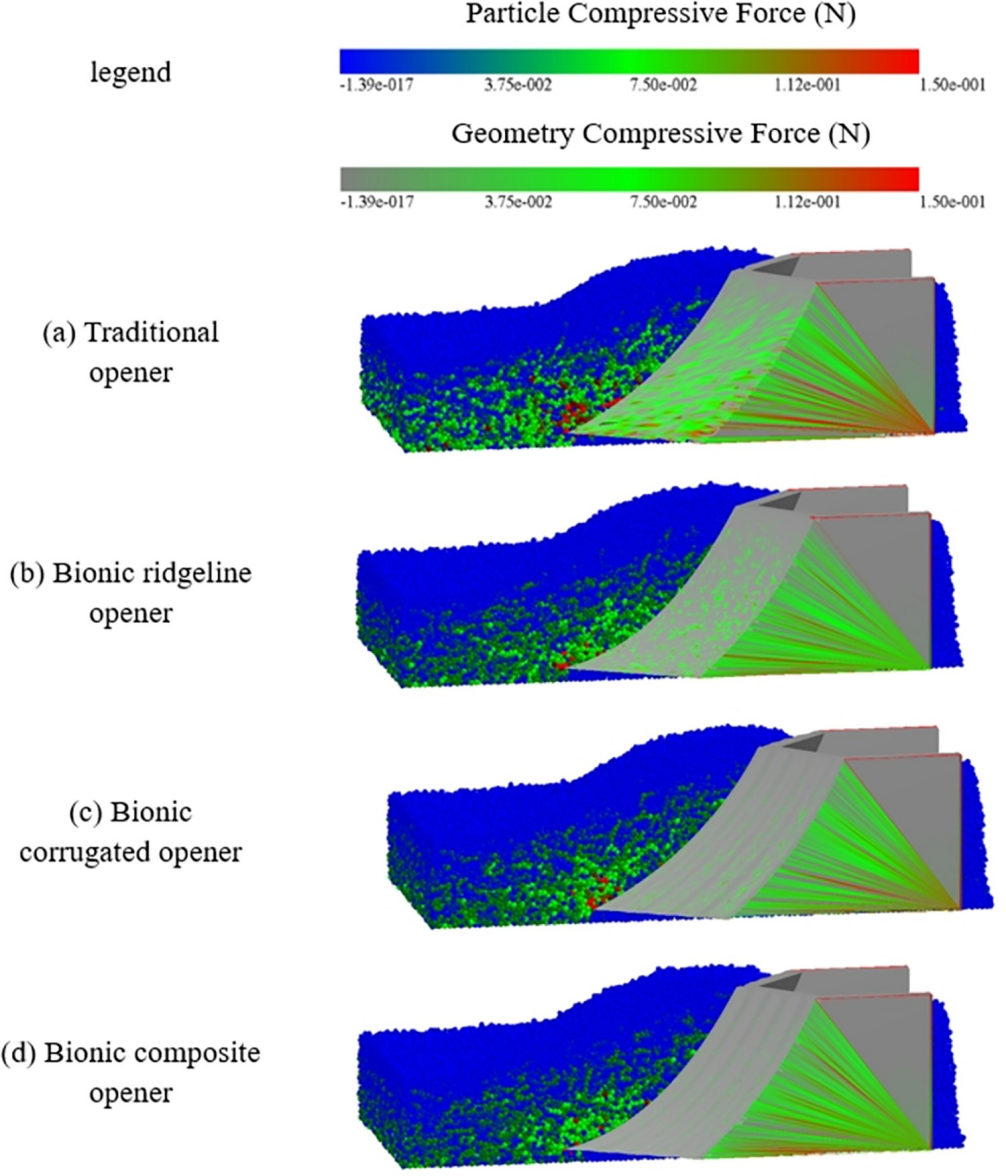
Contact force field diagram of opener and soil particles.

Particle contact force field analysis was performed in the ditching stable stage (2s). Fig 19 shows the contact force field between four openers and soil particles in the simulation process.

It can be seen from the force field diagram that the soil stress of the bionic ridgeline opener is small in the position where the core share is inserted into the soil, the force decreases uniformly along the ridgeline of the core. The traditional opener has a large force on the soil in the front part of the core, the force range is also large. The corrugated structure of the bionic corrugated opener causes the core surface to enter the soil part to have a certain range of disturbance to the soil, but the force field is small. The corrugated structure of the bionic corrugated opener causes the core surface to have a certain range of disturbance to the soil, but the force field is small. The corrugated structure can reduce the ditching resistance and have the effect of small range of loose soil and desorption. The bionic composite opener has the smallest force field and is evenly distributed uniformly along the core surface, including the advantages of drag reduction and loose soil and desorption. It can be seen from the force field diagram of the opener core surface; the corrugated structure can greatly relieve the core surface force and improve the ditching strength.

## Conclusions

1. According to the surface curve of the head part of the earthworm, three kinds of bionic opener were designed.
2. When the simulation is carried out at a ditching speed of 300 mm/s and a ditching depth of 30 mm, 60 mm and 90 mm, the drag reduction effect of the three bionic openers increases with the increase of the ditching depth. When the simulation is carried out at a ditching depth of 90 mm and a ditching speed of 50 mm/s, 100 mm/s, 200 mm/s, 300 mm/s, 600 mm/s and 900 mm/s, the drag reduction effect of the three bionic openers has different trends with the increase of the ditching speed. The bionic composite opener reached the highest drag reduction rate of all bionic openers when the depth is 60 mm, the speed is 100 mm/s, the value is 9.08%. The results showed that the order of ditching resistance from large to small is traditional opener, bionic corrugated opener, bionic ridgeline opener, bionic composite opener.
3. With the same ditching speed, the drag reduction effect of the three bionic openers increases with the increase of the ditching depth, During the process of increasing the depth from 30 mm to 60 mm and 90 mm, the ditching resistance of the traditional opener increased from 11.56 N to 28.32 N and 48.61 N as well as the maximum drag reduction ratio increased from 5.58% to 7.20% and 8.93% for the bionic omposite opener. With the same ditching depth, the bionic composite opener reached the highest drag reduction rate of all bionic openers when the speed is 100 mm/s, the value is 9.08%.
4. Three bionic openers have smaller ditch width and soil disturbance than the traditional opener, have better soil backing effect. The bionic ridgeline opener has a better penetration performance because the rake angle increases gradually and smoothly along the core ridgeline. The corrugated structure can guide the particles to run in the vertical direction, increase the ditch height and reduce the ditch width, relieve the force applied by the particles to the surface of the core from the tip of the core to the entire core surface. The corrugated structure creates a gap between the surface of the core and the particles, reducing the number of contact and contact area of the particles. The force field of the bionic composite opener is the smallest, the soil disturbance of the core surface is small and evenly distributed along the core surface.
5. The bionic opener has low ditching resistance and small ditching width, which satisfies the design requirements of the core opener, provides a theoretical basis for the design optimization of the furrow opener.

## Supporting information

S1 Data (DOCX)

S2 Data (XLSX)

S3 Data (XLSX)

S4 Data (XLSX)

S5 Data (XLSX)
