# Supplementary material for: Mesoscopic analysis of drag reduction performance of bionic furrow opener based on the discrete element method
Source: PLoS One. 2023 Nov 3;18(11):e0293750. doi: 10.1371/journal.pone.0293750 (PMC10624314; doi:10.1371/journal.pone.0293750)
Supplement: S1 Data — (DOCX) [file pone.0293750.s001.docx]

Table 1. Particle size distribution

| Particle diameter /mm | Above 3 | 2-3 | 1-2 | 1-0.5 | Below 0.5 |
| --- | --- | --- | --- | --- | --- |
| Particle proportion /% | 2.49 | 40.46 | 24.39 | 16.30 | 16.36 |

Table 2. Soil parameters

|  | Elastic modulus(MPa) | Poisson’s ratio | Shear modulus(MPa) |
| --- | --- | --- | --- |
| Experiment result | 2.92 | 0.38 | 1.05 |

Table 3. Soil parameters comparison

|  | Experimental Value | Simulation Value | Relative Error |
| --- | --- | --- | --- |
| Stacking Angle(º) | 35.9 | 36.5 | 1.6% |
| Collision Recovery Coefficient | 0.12 | 0.13 | 7.6% |
| Static Friction Coefficient | 0.37 | 0.40 | 7.5% |
| Rolling Friction Coefficient | 0.24 | 0.22 | 9% |
